# The value of forceps biopsy and core needle biopsy in prediction of pathologic complete remission in locally advanced rectal cancer treated with neoadjuvant chemoradiotherapy

**DOI:** 10.18632/oncotarget.5287

**Published:** 2015-09-18

**Authors:** Jing-Hua Tang, Xin An, Xi Lin, Yuan-Hong Gao, Guo-Chen Liu, Ling-Heng Kong, Zhi-Zhong Pan, Pei-Rong Ding

**Affiliations:** ^1^ State Key Laboratory of Oncology in South China, Collaborative Innovation Center for Cancer Medicine, Guangzhou, China; ^2^ Departments of Colorectal Surgery, Sun Yat-sen University Cancer Center, Guangzhou, China; ^3^ Departments of Medical Oncology, Sun Yat-sen University Cancer Center, Guangzhou, China; ^4^ Departments of Ultrasound, Sun Yat-sen University Cancer Center, Guangzhou, China; ^5^ Departments of Radiation Oncology, Sun Yat-sen University Cancer Center, Guangzhou, China

**Keywords:** forceps biopsy, core needle biopsy, locally advanced rectal cancer, neoadjuvant chemoradiotherapy

## Abstract

Patients with pathological complete remission (pCR) after treated with neoadjuvant chemoradiotherapy (nCRT) have better long-term outcome and may receive conservative treatments in locally advanced rectal cancer (LARC). The study aimed to evaluate the value of forceps biopsy and core needle biopsy in prediction of pCR in LARC treated with nCRT. In total, 120patients entered this study. Sixty-one consecutive patients received preoperative forceps biopsy during endoscopic examination. *Ex vivo* core needle biopsy was performed in resected specimens of another 43 consecutive patients. The accuracy for *ex vivo* core needle biopsy was significantly higher than forceps biopsy (76.7% *vs*. 36.1%; *p* < 0.001). The sensitivity for *ex vivo* core needle biopsy was significantly lower in good responder (TRG 3) than poor responder (TRG ≤ 2) (52.9% *vs.* 94.1%; *p* = 0.017). *In vivo* core needle biopsy was further performed in 16 patients with good response. Eleven patients had residual cancer cells in final resected specimens, among whom 4 (36.4%) patients were biopsy positive. In conclusion, routine forceps biopsy was of limited value in identifying pCR after nCRT. Although core needle biopsy might further identify a subset of patients with residual cancer cells, the accuracy was not substantially increased in good responders.

## INTRODUCTION

Neoadjuvant chemoradiotherapy (nCRT) followed by total mesorectal excision (TEM) and systemic chemotherapy is the standard treatment for patients with locally advanced rectal cancer (LARC) [1]. With this approach, 15–36% of patients have no residual viable cancer cells at pathological examination, referred as pathological complete remission (pCR), and these patients have better long-term outcome [2, 3]. Studies suggest that conservative approaches, such as local excision or even avoidance of surgery, might be alternative choices without compromising the oncologic outcome for patients with pCR [4, 5].

To date, there are still no reliable methods to identify patients with pCR before radical surgery. As a result, clinical complete response (cCR), defined as no clinical detectable tumor by physical examination, endoscopic evaluation, and imaging, is designed as a surrogate endpoint for pCR [4, 6]. However, the concordance between cCR and pCR varies from 22% to 96% in different reports [7–10], which questions the clinical value of such strategies. Therefore, it is of crucial significance to develop novel strategy to predict pCR for patients after nCRT in LARC.

Forceps biopsy and core needle biopsy are frequently used for the diagnosis of malignant tumors. Core needle biopsy is considered to be more accurate especially in tumor beneath epithelium which is a common phenomenon in LARC after neoadjuvant CRT. So far, there is no study reporting its value in evaluating pCR in the patients treated with neoadjuvant CRT. This study aimed to evaluate the value of forceps biopsy and core needle biopsy in predicting pCR in LARC after nCRT.

## RESULTS

### Patients’ demographics

Sixty-one patients received endoscopic forceps biopsy, among whom 12 (19.7%) patients achieved pCR. *Ex vivo* core needle biopsy was performed in 43 patients, among whom 8 (18.6%) achieved pCR. No statistically significant differences in the baseline characteristics were found between patients undergoing forceps biopsy and *ex vivo* core needle biopsy, except the interval between radiotherapy (RT) completion and biopsy (Table 1). *In vivo* core needle biopsy was performed in 16 patients with good response after CRT, and 5 (31.3%) patients achieved pCR (Table 2). There was no overlapping between different groups.

**Table 1:** Demographics of patients according to different biopsy approaches

Demographics	Forceps biopsy(*n* = 61)	*Ex vivo* core needle biopsy (*n* = 43)	*p* value
Age, years	54 (25–74)	55 (28–84)	0.747
Gender (female/male)	16/45	18/25	0.137
Distance from anal verge, cm	6.5 (1.0–13.0)	5.3 (0.5–12.0)	0.056
Interval between RT and biopsy, days	40.25 (21–83)	54.5 (29–80)	**<0.001**
Interval between RT and surgery, days	56.9 (42–82)	54.5 (29–80)	0.287
Clinical T stage			
cT2	6 (9.8)	3 (7.0)	0.809
cT3	32 (52.5)	26 (60.5)	
cT4	23 (37.7)	14 (32.6)	
Clinical N stage			
cN0	21 (34.4)	16 (37.2)	0.836
cN+	40 (65.6)	27 (62.8)	
Pathologic T stage			
ypT0	12 (19.7)	9(20.9)	0.701
ypTis	1 (1.6)	1(2.3)	
ypT1	2 (3.3)	0(0)	
ypT2	12 (19.7)	5(11.6)	
ypT3	30 (49.2)	27(62.8)	
ypT4	4 (6.6)	1(2.3)	
Pathologic N stage			
ypN0	50 (82.0)	34 (79.1)	0.802
ypN+	11 (18.0)	9 (20.9)	
Tumor regression grade(TRG)			
≤2	16 (26.2)	17 (39.5)	0.344
3	33 (54.1)	17 (39.5)	
4	12 (19.7)	9 (20.9)	

Data are given as n (%) or mean (range).

**Table 2:** Demographics of patients underwent *in vivo* core needle biopsy

Demographics	*In vivo* core needle biopsy (*n* = 16)
Age, y	58.5 (40–82)
Gender (female/male)	8/8
Distance from anal verge, cm	4.3 (2.0–8.0)
Interval between RT and biopsy, days	44.7 (29–67)
Interval between RT and surgery, days	56.5 (35–69)
Clinical T stage	
cT2	1 (6.3)
cT3	8 (50.0)
cT4	7 (43.8)
Clinical N stage	
cN0	3 (18.8)
cN+	13 (81.3)
Pathologic T stage	
ypT0	5 (31.3)
ypT2	2 (12.5)
ypT3	5 (31.3)
ypT4	4 (25)
Pathologic N stage	
ypN0	12 (75)
ypN+	4 (25)
Tumor regression grade (TRG)	
≤2	4 (25.0)
3	7 (43.8)
4	5 (31.3)

Data are given as n (%) or mean (range).

### Diagnostic performance of endoscopic forceps biopsy

Sixty-one patients received endoscopic forceps biopsy, among whom 51 patients were negative at biopsy and only 12 patients had no viable cancer cells found in the final resected specimens. The negative predictive value referred as the predictive value of pCR was 23.5%. Forty-nine patients had residual cancer cells in the final surgical specimens, among whom 10 patients were biopsy positive. The sensitivity was 20.4%, and the specificity was 100%. The overall accuracy was 36.1%. The result of histopathological findings of forceps biopsy compared with the surgical specimen was listed in Table 3.

**Table 3:** The result of histopathological findings of forceps biopsy compared with the surgical specimen

Forceps biopsy	Surgical specimen		Total
	No-pCR	pCR	
Tumor (+)	10	0	10
Tumor (−)	39	12	51
Total	49	12	61

Sensitivity = 20.4%; Specificity = 100%; Positive predictive value = 100%; negative predictive value = 23.5%; accuracy = 36.1%

Of the 49 patients with residual cancer cells at resected specimen (non-pCR), 16 (32.7%) patients were TRG ≤ 2 and 33 (67.3%) patients were TRG = 3. The sensitivity was not significantly different between TRG ≤ 2 and TRG = 3 (31.3% *vs*. 15.2%; *p* = 0.351) (Table 4).

**Table 4:** Correlation between TRG and forceps biopsy findings

TRG	Forceps biopsy		*p* value
	Tumor (+)	Tumor (−)	
TRG ≤ 2	5	11	0.351
TRG = 3	5	28	

TRG: tumor regression grade

### Diagnostic performance of *ex vivo* core needle biopsy

*Ex vivo* core needle biopsy was performed in 43 resected specimens. Of the 18 patients with biopsy negative, 8 achieved pCR. The negative predictive value was 44.4%. Thirty-five patients had residual cancer cells in the final resected specimens, among whom 25 patients were positive at core needle biopsy. The sensitivity was 71.4%, and the specificity was 100%. The overall accuracy was 76.7%. Concordance between surgical specimen and *ex vivo* core needle biopsy samples was listed in Table 5.

**Table 5:** The result of histopathological findings of *ex vivo* core needle biopsy compared with the surgical specimen

*Ex vivo* core needle biopsy	Surgical specimen		Total
	No-pCR	pCR	
Tumor (+)	25	0	25
Tumor (−)	10	8	18
Total	35	8	43

Sensitivity = 71.4%; Specificity = 100%; Positive predictive value = 100%; negative predictive value = 44.4%; accuracy = 76.7%

Among 35 patients with residual cancer cells in the final resected specimens, 17 patients were TRG ≤ 2; 17 patients were TRG = 3; and 1 patient was pathological complete regression in primary tumor (ypT0 or TRG4) but had residual tumor cells in the lymph nodes (ypN+). The sensitivity was 94.1% with TRG ≤ 2 and 52.9% with TRG = 3. The sensitivity was significantly different between TRG ≤ 2 and TRG = 3 (*p* = 0.017) (Table 6).

**Table 6:** Correlation between TRG and *ex vivo* core needle biopsy

TRG	*ex vivo* core needle biopsy		*p* value
	Tumor (+)	Tumor (−)	
TRG ≤ 2	16	1	0.017
TRG = 3	9	8	

TRG: tumor regression grade

### Diagnostic performance of *in vivo* core needle biopsy

Sixteen patients with good response evaluated by digital rectal examination, colonoscopy and forceps biopsy, and MRI after nCRT received *in vivo* core needle biopsy. Residual cancer cells were detected in 11 (68.8%) patients at the final pathologic examination, among whom 4 patients were biopsy positive. The sensitivity was 36.4%, and the specificity was 100%. Of the 12 patients negative at *in vivo* core needle biopsy, 5 patients achieved pCR. The negative predictive value was 41.7%. The overall accuracy was 56.3% (Table 7). Except for some discomfort experienced during the biopsy, no serious procedure-related complications were observed in any of these 16 patients.

**Table 7:** The result of histopathological findings of *in vivo* core needle biopsy compared with the surgical specimen

*In vivo* core needle biopsy	Surgical specimen		Total
	No-pCR	pCR	
Tumor (+)	4	0	4
Tumor (−)	7	5	12
Total	11	5	16

Sensitivity = 36.4%; Specificity = 100%; Positive predictive value = 100%; negative predictive value = 41.7%; accuracy = 56.3%

## DISCUSSION

Our study demonstrates that forceps biopsy is an inaccurate method of identifying patients with pCR after nCRT. Although the accuracy of *ex vivo* core needle biopsy has been markedly improved compared to forceps biopsy, the accuracy of *in vivo* core needle biopsy is far from perfect in the patients with good response. To our knowledge, it is the first study to analyze the value of core needle biopsy in predicting pCR through *ex vivo* and *in vivo*. The findings of this study may have significant clinical implications because they highlight the limitation of forceps biopsy and core needle biopsy in prediction of pCR in LARC treated with nCRT.

In patients with negative forceps biopsy, only 23.5% patients achieved pCR, which is consistent with the previous reports showing that the negative predictive value for forceps biopsy was 21.4% and 36% [11, 12]. Similarly, in a Habr-Gama's study, the negative predictive value was 21% for those with significant tumor downsizing [13]. The low accuracy might be attributed to the redistribution of the residual cancer cells after CRT. Duldulao MP et al revealed that residual cancer cells in rectal cancer after CRT preferentially located to the invasive front of the tumor, and at least 25% patients with cT2 tumors and more than 40% patients with cT3/4 tumors after CRT that did not achieve pCR would have no residual cancer cells in the mucosa or submucosa [14]. Dana M. Hayden and Fraser M. Smith reported that residual tumor cells were presented lateral and distal to the residual mucosal abnormalities in patients after neoadjuvant radiotherapy [15, 16]. The current study reinforces that forceps biopsy is of limited clinical value due to the inherent flaws of inability to obtain specimens deep to the muscularis or subserosa layer.

To date, there is no study reporting the value of core needle biopsy in predicting a pCR. Therefore, we hypothesized that core needle biopsy might improve the diagnostic performance. In the current study, we first confirmed the hypothesis by performing an *ex vivo* core needle biopsy in the tumor bed in the resected specimen. Our results showed that the overall accuracy was significantly improved compared to forceps biopsy (76.7% vs. 36.1%; *p* < 0.001). We further tested the hypothesis by *in vivo* core needle biopsy guided by ERUS in good responders. Although the method is able to identify additional 36.4% of patients with residual disease after ruling out patients with obvious non-pCR, the overall accuracy is far from perfect. These were not unexpected since a false-negative result was more likely to occur in patients with minimal residual cancer cells or good responders, whom the *in vivo* core needle biopsy aims to. Moreover, we found a correlation between tumor response and sensitivity of *ex vivo* core needle biopsy, and the sensitivity was significantly higher in patients with mild or moderate response (TRG ≤ 2) than patients with better response (TRG = 3). Fewer patients were TRG ≤ 2 in the group of *in vivo* core needle biopsy, which may contribute to the low accuracy. Meanwhile, it might be more difficult to determine the target lesion in ERUS than direct observation in *ex vivo* core needle biopsy.

Of note, one patient (11.1%) obtained *ex vivo* core needle biopsy achieved primary tumor regression (ypT0) but had residual tumor cells in the lymph nodes (ypN+). This incidence ranged from 2% to 17% in previous studies [17, 18]. The inability of those biopsy approaches or radical imagine techniques to accurately identify these patients might challenge the less aggressive strategy.

Limitations in this study should be mentioned. Several studies demonstrated that a prolonged interval to assess tumor response could maximize tumor regression and result in a higher chance of pCR [19, 20]. In this study, patients underwent *ex vivo* core needle biopsy nearly 2 weeks later than other approaches, which may account for some discrepancies. In addition, this study was performed in a single-institution and the selected population of *in vivo* core needle biopsy in good responders might also leaded to bias.

In conclusion, the current study showed that forceps biopsy was of limited clinical value in identifying patients with pCR after CRT. Although, core needle biopsy might further identify a subset of patients with residual cancer cells, the accuracy was not substantially increased in good responders.

## MATERIALS AND METHODS

### Patients

Between Nov. 2011 and Dec. 2013, a total of 120 patients with LARC treated with nCRT at Sun Yat-sen University Cancer Center were prospectively included in this study. This study was approved by the Institutional Ethical Review Boards of the Sun Yat-sen University Cancer Center, and performed in accordance with the ethical standards laid down in the Declaration of Helsinki. Written informed consent was obtained from each patient before enrollment in the trial. All patients were biopsy-proven adenocarcinoma. Clinical staging of local tumor was performed by Magnetic Resonance Image (MRI) and endoscopic ultrasound (EUS).

The total dosage of radiotherapy was 50 Gy consisted of 23 fractions of 2 Gy to clinical target volume and 2 fractions of 2 Gy to gross tumor volume. Patients received two cycles of modified XELOX (Oxaliplatin 100 mg/m^2^ d1, Capecitabine 1000 mg/m^2^ bid, d1–14) regimens on day 1–14 and day 21–34 concomitant to radiotherapy. The standard TME surgery was planned 6 to 8 weeks after completion of nCRT.

### Forceps biopsy, *ex vivo* core needle biopsy, and *in vivo* core needle biopsy

Firstly, 61 patients were consecutively enrolled into the forceps biopsy group to evaluate the value in prediction of pCR. These patients underwent forceps biopsies during the endoscopic examination using 2.8-mm biopsy forceps about 6 weeks after the completion of nCRT. At least 3 biopsy samples were taken from the suspicious sites from every patient.

To evaluate the possible role of core needle biopsy in the diagnosis of pCR, 43 patients were subsequently enrolled to the *ex vivo* core needle biopsy group before proceeding with the procedure *in vivo*. The standard TME surgery was performed 6 to 8 weeks after the end of nCRT. Once the specimen was removed and opened, the researcher performed an *ex vivo* core needle biopsy with an 18-gauge core needle direct to three representative regions of the tumor bed. In patients with suspected macroscopic complete response, the scar tissue was biopsied accordingly.

To validate the *ex vivo* findings and evaluate the safety of the procedure, core needle biopsy was further performed *in vivo* about 6 weeks after the completion of nCRT in 16 patients with good response. The definition of good response, determined by clinical examination, endoscopy and forceps biopsy, and MRI, was (1) substantial tumor downsizing (>30% reduction of the initial tumor size); (2) No obvious residual tumor or deep ulceration at endoscopy. A superficial ulcer or scar might be acceptable; and (3) negative forceps biopsies from the ulcer or former tumor location. Before the biopsy, patients received routine cleansing enema by 0.1% soap solution. Patients were examined initially with the ultrasound probe in the left lateral decubitus position. When hypoechoic area of the rectal wall was identified through ERUS and felt to represent the suspicious site of residual tumor, core needle biopsy would be performed under direct ultrasound guidance with an 18-gauge core needle. At least 3 samples were achieved in each patient.

### Pathologic assessment

All the biopsy samples were sent for routine pathological analysis and compared with the histopathological result of the resected specimens. Sensitivity, specificity, positive predictive value, negative predictive value, and accuracy were analyzed.

Pathological stage was determined according to the AJCC 7th edition [21]. Neoadjuvant CRT effect was evaluated using tumor regression grade (TRG) system by Dworak et al as follows [22]: Grade 0: no regression; Grade 1: dominant tumor mass with obvious fibrosis and/or vasculopathy; Grade 2: dominantly fibrotic changes with few tumor cells or groups (easy to find); Grade 3 (or nearly pCR): very few (difficult to find microscopically) tumor cells in fibrotic tissue with or without mucous substance; Grade 4: no tumor cells, only fibrotic mass (total regression or response).

### Statistical analysis

Statistical analysis was performed using the chi-square and Fisher's exact tests. A value of *p* < 0.05 was considered statistically significant. Statistical Package for the Social Sciences (SPSS)’ version 16.0 for Windows (SPSS, Inc. Chicago, IL) was used for all analysis.

## References

[1] Sauer R, Becker H, Hohenberger W, Rödel C, Wittekind C, Fietkau R, Martus P, Tschmelitsch J, Hager E, Hess CF, Karstens JH, Liersch T, Schmidberger H (2004). Preoperative versus postoperative chemoradiotherapy for rectal cancer. N Engl J Med.

[2] Maas M, Nelemans PJ, Valentini V, Das P, Rödel C, Kuo LJ, Calvo FA, García-Aguilar J, Glynne-Jones R, Haustermans K, Mohiuddin M, Pucciarelli S, Small W (2010). Long-term outcome in patients with a pathological complete response after chemoradiation for rectal cancer: a pooled analysis of individual patient data. Lancet Oncol.

[3] Gao YH, Zhang X, An X, Cai MY, Zeng ZF, Chen G, Kong LH, Lin JZ, Wan DS, Pan ZZ, Ding PR (2014). Oxaliplatin and Capecitabine concomitant with neoadjuvant radiotherapy and extended to the resting period in high risk locally advanced rectal cancer. Strahlenther Onkol.

[4] Maas M, Beets-Tan RG, Lambregts DM, Lammering G, Nelemans PJ, Engelen SM, van Dam RM, Jansen RL, Sosef M, Leijtens JW, Hulsewé KW, Buijsen J, Beets GL (2011). Wait-and-see policy for clinical complete responders after chemoradiation for rectal cancer. J Clin Oncol.

[5] Habr-Gama A, Perez RO, Nadalin W, Sabbaga J, Ribeiro U, Silva e Sousa AH, Campos FG, Kiss DR, Gama-Rodrigues J (2004). Operative versus nonoperative treatment for stage 0 distal rectal cancer following chemoradiation therapy: long-term results. Ann Surg.

[6] Habr-Gama A, Perez RO, Wynn G, Marks J, Kessler H, Gama-Rodrigues J (2010). Complete clinical response after neoadjuvant chemoradiation therapy for distal rectal cancer: characterization of clinical and endoscopic findings for standardization. Dis Colon Rectum.

[7] Guillem JG, Chessin DB, Shia J, Moore HG, Mazumdar M, Bernard B, Paty PB, Saltz L, Minsky BD, Weiser MR, Temple LK, Cohen AM, Wong WD (2005). Clinical examination following preoperative chemoradiation for rectal cancer is not a reliable surrogate end point. J Clin Oncol.

[8] Glynne-Jones R, Wallace M, Livingstone JIL, Meyrick-Thomas J (2008). Complete clinical response after preoperative chemoradiation in rectal cancer: Is a “Wait and See” policy justified?. Dis Colon Rectum.

[9] Hiotis SP, Weber SM, Cohen AM, Minsky BD, Paty PB, Guillem JG, Wagman R, Saltz LB, Wong WD (2002). Assessing the predictive value of clinical complete response to neoadjuvant therapy for rectal cancer: an analysis of 488 patients. J Am Coll Surg.

[10] Perez RO, Habr-Gama A, Gama-Rodrigues J, Proscurshim I, Julião GP, Lynn P, Ono CR, Campos FG, Silva e Sousa AH, Imperiale AR, Nahas SC, Buchpiguel CA (2012). Accuracy of positron emission tomography/computed tomography and clinical assessment in the detection of complete rectal tumor regression after neoadjuvant chemoradiation Long-Term Results of a Prospective Trial (National Clinical Trial 00254683). Cancer.

[11] Kuo LJ, Chiou JF, Tai CJ, Chang CC, Kung CH, Lin SE, Hung CS, Wang W, Tam KW, Lee HC, Liang HH, Chang YJ, Wei PL (2012). Can we predict pathologic complete response before surgery for locally advanced rectal cancer treated with preoperative chemoradiation therapy?. Int J Colorectal Dis.

[12] Meterissian S, Skibber J, Rich T, Roubein L, Ajani J, Cleary K, Ota DM (1994). Patterns of residual disease after preoperative chemoradiation in ultrasound T3 rectal carcinoma. Ann Surg Oncol.

[13] Perez RO, Habr-Gama A, Pereira GV, Lynn PB, Alves PA, Proscurshim I, Rawet V, Gama-Rodrigues J (2012). Role of biopsies in patients with residual rectal cancer following neoadjuvant chemoradiation after downsizing: can they rule out persisting cancer?. Colorectal Dis.

[14] Duldulao MP, Lee W, Streja L, Chu P, Li W, Chen Z, Kim J, Garcia-Aguilar J (2013). Distribution of residual cancer cells in the bowel wall after neoadjuvant chemoradiation in patients with rectal cancer. Dis Colon Rectum.

[15] Hayden DM, Jakate S, Pinzon MC, Giusto D, Francescatti AB, Brand MI, Saclarides TJ (2012). Tumor scatter after neoadjuvant therapy for rectal cancer: are we dealing with an invisible margin?. Dis Colon Rectum.

[16] Smith FM, Wiland H, Mace A, Pai RK, Kalady MF (2014). Depth and Lateral Spread of Microscopic Residual Rectal Cancer after Neoadjuvant Chemoradiation: Implications for Treatment Decisions. Colorectal Dis.

[17] Pucciarelli S, Capirci C, Emanuele U, Toppan P, Friso ML, Pennelli GM, Crepaldi G, Pasetto L, Nitti D, Lise M (2005). Relationship between pathologic T-stage and nodal metastasis after preoperative chemoradiotherapy for locally advanced rectal cancer. Ann Surg Oncol.

[18] Hughes R, Glynne-Jones R, Grainger J, Richman P, Makris A, Harrison M, Ashford R, Harrison RA, Livingstone JI, McDonald PJ, Meyrick Thomas J, Mitchell IC, Northover JM (2006). Can pathological complete response in the primary tumor following pre-operative pelvic chemoradiotherapy for T3-T4 rectal cancer predict for sterilization of pelvic lymph nodes, a low risk of local recurrence and the appropriateness of local excision?. Int J Colorectal Dis.

[19] Sloothaak DA, Geijsen DE, van Leersum NJ, Punt CJ, Buskens CJ, Bemelman WA, Tanis PJ (2013). Optimal time interval between neoadjuvant chemoradiotherapy and surgery for rectal cancer. Br J Surg.

[20] Moore HG, Gittleman AE, Minsky BD, Wong D, Paty PB, Weiser M, Temple L, Saltz L, Shia J, Guillem JG (2004). Rate of pathologic complete response with increased interval between preoperative combined modality therapy and rectal cancer resection. Dis Colon Rectum.

[21] Edge SB, Compton CC (2010). The American Joint Committee on Cancer: the 7th edition of the AJCC cancer staging manual and the future of TNM. Ann Surg Oncol.

[22] Dworak O, Keilholz L, Hoffmann A (1997). Pathological features of rectal cancer after preoperative radiochemotherapy. Int J Colorectal Dis.

